# Pregnancy exposure to individual phthalate concentrations and their mixtures in relation to pediatric serum antibody response

**DOI:** 10.1007/s10654-026-01387-1

**Published:** 2026-03-12

**Authors:** Marina Oktapodas Feiler, Michele Salerno, Sally A. Quataert, Martha M. Tellez-Rojo, Hector Lamadrid-Figueroa, Guadalupe Estrada, Robert O. Wright, Todd A. Jusko, Elena Colicino

**Affiliations:** 1 Department of Epidemiology and Environmental Health, School of Public Health and Health Professions, State University of New York at Buffalo, Buffalo, USA; 2 Department of Environmental Medicine, Icahn School of Medicine at Mount Sinai, New York, NY USA; 3 Data Science Unit, Fondazione IRCCS Fondazione Istituto Nazionale dei Tumori di Milano, Milan, Italy; 4 Department of Microbiology and Immunology, School of Medicine and Dentistry, University of Rochester, Rochester, USA; 5 Center for Nutrition and Health Research, National Institute of Public Health, Cuernavaca, Morelos Mexico; 6 Department of Perinatal Health, National Institute of Public Health, Cuernavaca, Morelos Mexico; 7 Department of Immunobiochemistry, National Institute of Perinatology, Mexico City, Mexico; 8 Department of Public Health Sciences, School of Medicine and Dentistry, University of Rochester, Rochester, USA; 9 Department of Biosciences, University of Milan, Milan, Italy

**Keywords:** Plasticizers, Vaccine, Gestation, In-utero, MMR, DTP, Sex-differences

## Abstract

The objective of this study was to estimate the association between gestational phthalate metabolite concentrations and mixtures and pediatric antibody response at 4-years of age to common childhood vaccines, while also exploring sexually dimorphic effects. This study utilized data from the Programming, Research, Obesity, and Social Stressors (PROGRESS) Study, an ongoing, longitudinal cohort of mother-child pairs residing in Mexico City. Fifteen phthalate metabolites were measured in spot urine samples collected from mothers during their second and third trimesters. Children have been regularly followed, with data collection on lifestyle, clinical, socio-economic, and demographic factors, and archived biologic specimen. IgG-specific antibody serum levels to measles, mumps, rubella, diphtheria, tetanus, and pertussis were quantified from children at the mean 4.7 years of age. Linear regression models, with log2 transformation of both the outcome and exposure variables, estimated the association between individual phthalate metabolites and antibody concentrations. Phthalate mixtures were analyzed using the Quantile G-Computational approach and Bayesian Kernel Machine regression. All analyses were also sex-stratified to investigate sexually dimorphic effects. The present analysis included 362 mother-child pairs. During the second trimester of pregnancy, a doubling increase of mono-2-ethyl-5-carboxypentyl terephthalate (MECPTP) concentrations was associated with a 6.98% decrease (95% CI: − 11.68%, − 2.04%) in diphtheria and a 2.57% decrease (95% CI: − 4.74%, − 0.35%) in mumps antibody levels, respectively. No statistically significant sex-differences were observed. Mixtures analyses did not reach statistical significance but observed similar associations with MECPTP. Concentrations of MECPTP, a replacement phthalate, were negatively associated with anti-diphtheria antibody levels in Hispanic children indicating a potential detrimental effect of newer alternative phthalates on pediatric health.

**Supplementary Information:**

The online version contains supplementary material available at 10.1007/s10654-026-01387-1.

## Introduction

Immune responses to routine childhood vaccination are a critical indicator of immune competence in early life [1]. While most children mount protective antibody responses, variability exists, and impaired vaccine response has been linked to increased susceptibility to infectious disease [2]. Identifying modifiable early-life factors that influence vaccine-derived immunity remains an important epidemiologic objective.

Environmental chemical exposures during gestation have been implicated in altered immune outcomes, consistent with the Developmental Origins of Health and Disease (DOHaD) framework [3]. Epidemiologic studies have reported associations between prenatal or early-life exposure to polychlorinated biphenyls (PCBs), poly- and perfluoroalkyl substances (PFAS), and metals and reduced antibody responses to childhood vaccines [4–12]. In contrast, evidence linking gestational exposure to phthalates, a ubiquitous class of endocrine-disrupting chemicals, to vaccine-related immune outcomes in humans is sparse. To date, only one small prospective study (*N* = 81) has examined gestational and postnatal phthalate exposure in relation to childhood vaccine response among a Taiwanese cohort of mother-child pairs, reporting inverse associations between select phthalate metabolites and hepatitis B antibody levels during adolescence [12]. No prior studies have evaluated associations with other routine childhood vaccines, nor have they assessed phthalate mixtures or potential sex-specific effects.

Phthalates, are widely used as plasticizers, stabilizers, and solvents in consumer products, resulting in abundant exposure among pregnant individuals and children. Phthalates can be found in children’s toys, medical devices, food packaging, and cosmetics like lotions and sunscreens [13, 14], as well as household, commercial, industrial, and pharmaceutical uses making risk of exposure universal [14]. Traditional phthalates such as di-2-ethylhexyl phthalate (DEHP), dibutyl phthalate (DBP), diethyl phthalate (DEP), and benzyl butyl phthalate (BBP) are widely found in the consumer products and increasingly being replaced by alternative plasticizers, including diisononyl cyclohexane-1,2-dicarboxylate (DINCH) and di-2-ethylhexyl terephthalate (DEHTP) [15, 16]. Although population-level declines in some traditional phthalates have been observed, biomonitoring studies indicate rising exposure to replacement phthalates, including among pregnant populations in settings with limited regulatory controls [17]. Previously published work in Mexico observed no change in traditional phthalates overtime, however, increases in alternative phthalate metabolites were observed, specifically for DEHTP and mono-2-ethyl-5-carboxypentyl terephthalate (MECPTP) [18]. MECPTP is a metabolite of DEHTP which has been used to replace DEHP, due to a suggestive decreased toxicity of DEHTP [19]. Unfortunately, emerging data suggests these alternatives are associated with similar health concerns as traditional phthalates, warranting further monitoring and examination of these chemicals [20].

Children are readily exposed to phthalates *in utero* and early life due to their use in consumer products [21]. According to the US Consumer Product Safety Commission there are some regulations on phthalate usage in the US concerning children’s consumer items [22]. Child toys or care articles are not permitted concentrations over 0.1% weight for eight common phthalates [22]. There may also be partial transplacental transfer of these phthalates suggesting gestational exposure as a recent study observed modest correlations of *r* = 0.25–0.36 of different phthalate metabolites in maternal urine and amniotic fluid samples [23]. Furthermore, previously published distributions of exposure from the cohort used in the present analysis in Mexico City reported annual increases of 30% (95% CI: 23–39%) in phthalate exposures from 2007 to 2010 [18]. Unfortunately, unlike the US, Mexico has no current regulations on phthalate usage in commercial products, evident in the increasing exposure trends found in this population of mothers in Mexico City [18]

Several methodological gaps remain in the existing literature. Most human studies rely on a single gestational exposure measurement, despite evidence that phthalate concentrations can vary across pregnancy, suggesting potential sensitivity to exposure timing [24, 25]. Additionally, phthalates co-occur as complex mixtures yet no prior studies have evaluated joint exposure effects in relation to vaccine response [26, 27]. Finally, emerging epidemiologic evidence suggests sex-specific vulnerability to prenatal environmental exposures [28, 29], but sexually dimorphic effects have not been examined for phthalates and immune outcomes.

The objective of the present study was to estimate the association between gestational phthalate metabolite urinary concentrations and their mixtures and pediatric antibody response at 4-years of age to common childhood vaccines including the measles, mumps, rubella (MMR) and diphtheria, tetanus, pertussis (DTP) vaccines. We further examined trimester-specific exposure windows and explored potential sex differences in these associations.

## Methods

### Study population

The Programming, Research, Obesity, and Social Stressors (PROGRESS) Study is an ongoing, longitudinal cohort of 948 mother-child pairs residing in Mexico City. Recruitment and enrollment began in the mothers’ second trimester among women whose prenatal visits and subsequent deliveries were at Hospital de Ginecología y Obsetricia between 2007 and 2012 [30, 31]. Women were eligible if they were at least 18 years of age, less than 20 weeks gestation at time of recruitment, planning to stay in Mexico City for the next 3 years, and had access to a telephone [32]. Mothers were excluded during pregnancy if they (1) lived in a household outside the metropolitan area, (2) suffered from psychiatric diseases, (3) used steroids or anti-epilepsy drugs, (4) consumed alcohol daily, or (5) had heart or kidney disease, and after birth, mothers were excluded at delivery if (1) their newborns had serious birth defects, and (2) they delivered multiples [30]. All participating mothers received a detailed explanation of the study and its procedures [30, 31]. Children have been regularly followed, with data collection on lifestyle, clinical, socio-economic, and demographic factors, and archived biologic specimens (i.e., blood, urine, saliva) [31]. The study protocols were approved by the institutional review boards at the Harvard School of Public Health, Icahn School of Medicine at Mount Sinai and the Research, Ethics in Research and Biosafety Committees in the Mexican National Institute of Public Health and National Institute of Perinatology.

**Fig. 1:**
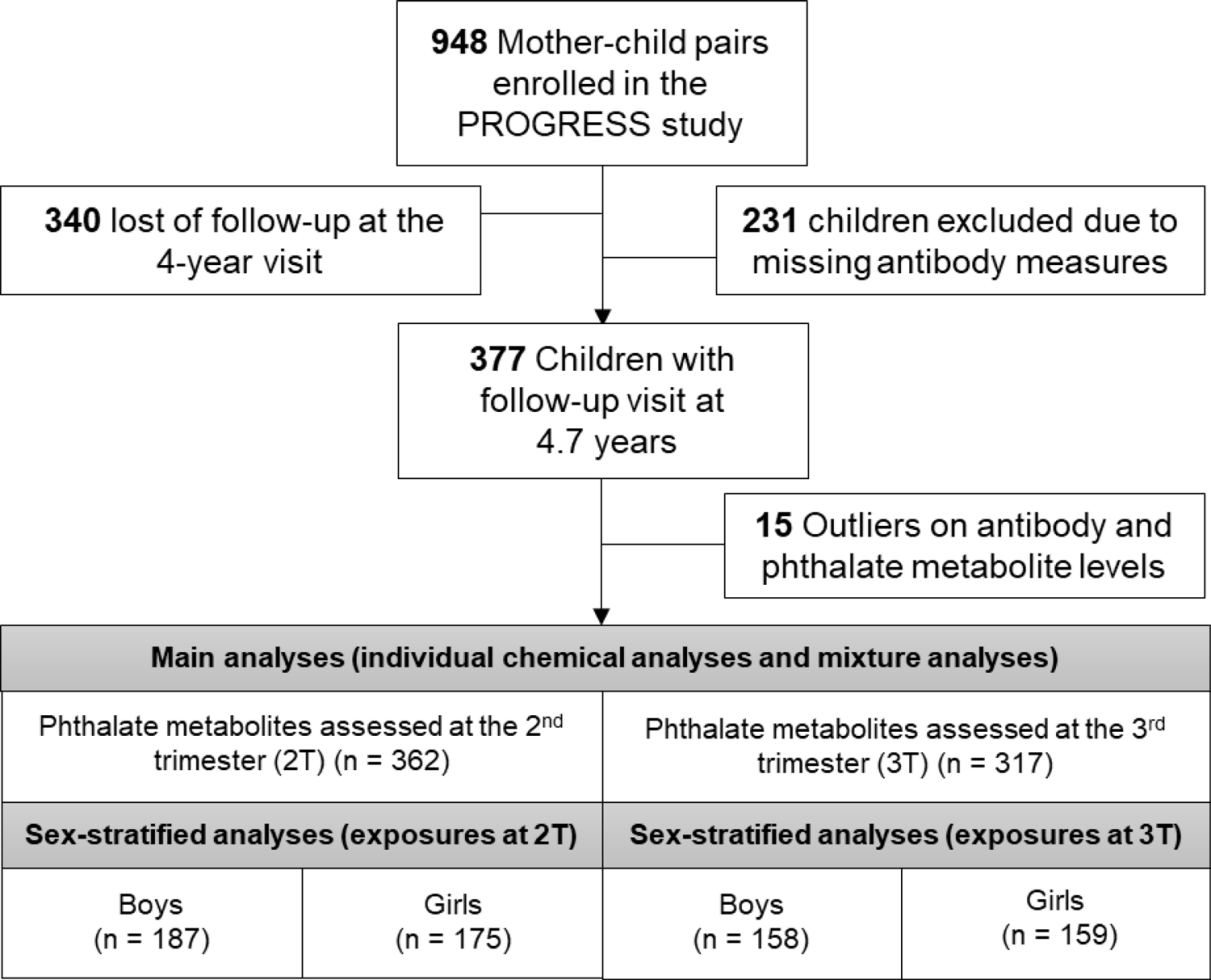
Overview of participant exclusions due to missing information on phthalate metabolites, antibody levels, and covariate data

Mother-child pairs with missing data on antibody responses and urinary gestational phthalate metabolite concentrations were excluded (Fig. 1). In addition, outliers detected using the interquartile rule with a k factor of 4 for antibody and phthalate metabolites variables were removed (*n* = 15), with a final sample size of *n* = 362 (Fig. 1). This more conservative threshold preserves the natural variability of biological data, reduces the possibility of eliminating plausible observations, and enhances sample size. Participants included in the study did not differ from those excluded (Table S1).

### Gestational urinary phthalate metabolite measurement

Spot urine samples were collected from mothers during the second and third trimesters in polypropylene specimen collection cups, and 2mL aliquots were stored at −80˚C. Quantification of 15 phthalate metabolites was conducted at the Centers for Disease Control and Prevention (CDC) using isotope dilution high-performance liquid chromatography coupled with tandem mass spectrometry [18]. These 15 metabolites included: mono-2-ethyl-5-carboxypentyl terephthalate (MECPTP), monobenzyl phthalate (MBzP2), mono carboxyisononyl phthalate (MCNP), monooxononyl phthalate (MONP), mono carboxisooctyl phthalate (MCOP), mono-2-ethyl-5-carboxypentyl phthalate (MECPP), mono–2-ethyl-5-oxohexyl phthalate (MEOHP), mono-2-ethyl-5-hydroxyhexyl phthalate (MEHHP), mono-2-ethylhexyl phthalate (MEHP), mono-3-carboxypropyl phthalate (MCPP), mono-hydroxyisobutyl phthalate (MHiBP), mono-hydroxybutyl phthalate (MHBP), mono-isobutyl phthalate (MiBP), mono-n-butyl phthalate (mBP), monoethyl phthalate (MEP). The limits of detection (LODs) ranged from 0.2 to 1.2 ng/mL, depending on the metabolite. Quality control measures included following analytical quality control protocols of the CDC laboratory and using a pool of anonymous human adult urine (BioIVT, NY, US) as a blinded replicate randomly inserted throughout the study samples [18]. Variations were small compared to the population distribution [18]. Most samples (100 − 95%) contained detectable concentrations of several of 15 metabolites measured (Table S2). Concentrations below the detection limit (< LOD) were replaced with the LOD/$\surd\left(2\right)$.

Specific gravity was measured at room temperature with a handheld digital refractometer (AR200, Reichert Technologies, Buffalo, NY) at the Centers for Disease Control and Prevention (CDC). For samples from the second and third trimester visit with missing specific gravity values (*n* = 27; *n* = 47), the respective median value was used. Phthalate metabolite concentrations in urine were adjusted for specific gravity using the following formula:

$${P}_{c}=P\times\left(\frac{mean\left(SG\right)-1}{SG-1}\right)$$

where Pc is the specific gravity–adjusted concentration (micrograms per liter), P is the observed concentration (micrograms per liter), and SG is the urine sample’s specific gravity. To minimize skewness, all metabolite concentrations were log2-transformed.

In the analysis, all metabolites were evaluated separately for the second and third trimesters.

### IgG-specific antibody response

Anti-measles, anti-mumps, anti-rubella, anti-diphtheria, anti-tetanus, and anti-pertussis IgG-specific serum levels were quantified using a multiplexed magnetic bead array assay (Luminex Technology) in plasma samples collected at 4.7 years of age [33]. Given high vaccination rates due to daycare and school policies in Mexico and low community exposure, serum antibody levels are likely attributable to vaccination. Blood samples were previously collected on all children during their four-year follow-up visit. Antibody levels were measured at the Rochester Human Immunology Center (RHIC) Core Laboratory at the University of Rochester (UR) under the supervision of Dr. Sally Quataert (Director).

### Additional covariates

All covariates were selected a priori based on team expertise and literature review and were examined as potential confounders utilizing a directed acyclic graph (DAG) [34]. Selected covariates from the DAG were all collected in questionnaires in PROGRESS, including children’s age (years), children’s sex, and maternal socioeconomic status based on the guidelines of the Mexican Association of Public Opinion and Research Agencies (AMAI) [35]. The AMAI index considered a total of 13 factors including level of education of the head of household, number of bedrooms in the home, number of full bathrooms, access to internet, number of employed persons living in the household, number of appliances in the household, car ownership, and additional household related factors. The index classifies families into 6 levels, which were further collapsed into three categories: low, medium, and high.

### Statistical analysis

Both the phthalate metabolite exposures and the outcome variables were log2-transformed to facilitate the interpretation of results and meet the normality assumptions of the models. All results were reported as the percent change (%) in mean antibody levels for a doubling increase of individual phthalate metabolite concentration or a decile increase in the phthalate metabolite quantile g-comp mixture (i.e. (2^b^ – 1) × 100%, where b represents the coefficient of the outcome association with the individual metabolite or the phthalate metabolite mixture, respectively). All analyses were adjusted for child age, sex, and family socioeconomic status and were conducted using R, version 4.3.1.

#### Individual phthalate metabolite analysis

Linear regression models estimated the association between individual phthalate metabolites and antibody concentrations for the MMR and DTP vaccines. All results were corrected for multiple testing using a 10% False Discovery Rate (FDR).

#### Quantile G-computational approach

We applied the quantile g-computation approach to analyze the association between antibody levels and phthalate metabolite mixtures, including all individual phthalate metabolites. This approach evaluated the linear and additive associations between the overall exposure biomarker mixture and the outcomes. The overall mixture was a linear weighted combination of phthalate metabolites, where weights described the relative importance of the individual phthalate metabolite to the mixture. All phthalate metabolite exposures were ranked in deciles to facilitate the results interpretation. To ensure robust estimates, the analysis employed 1,000 bootstrap iterations. The analyses were performed using the qgcomp package in R (version 4.3.1).

#### Bayesian Kernel machine regression

We also applied the Bayesian Kernel Machine Regression (BKMR) method to analyze the association between antibody response concentrations and phthalate metabolite mixture exposure. This approach complements the quantile G-computation approach, as it considers synergies among metabolites and non-linear metabolite-outcome associations. Phthalate metabolite concentrations were standardized to meet the model assumptions, and the model was fit using the kmbayes function with 10,000 iterations and the enabled variable selection option. We used the default probability priors and hyperpriors, i.e., normal distributions for the regression coefficients, inverse-gamma distribution for the residual variance, and uniform distributions for kernel hyperparameters. Convergence was assessed by the inspection of trace plots for each coefficient, variance, and the posterior inclusion probabilities confirmed to show good mixing and stability of the algorithm. The analysis was performed using the bkmr package in R (version 4.3.1).

#### Sex stratified analysis

Phthalates metabolites have been shown to have sexually dimorphic associations in relation to pediatric outcomes, so we considered sex-stratified analysis for models showing statistically significant associations in the overall population.

## Results

This study included 362 PROGRESS children with a mean age of 4.75 years (SD = 0.7), similarly distributed by sex (48% girls, 52% boys. Table 1; Figure S1). The age distribution was consistent across boys and girls, with most children being 4 or 5 years old (40% and 48%, respectively). Approximately half of the sample (51%) was low SES, and the SES distribution was similar between boys and girls (*p* = 0.91). In terms of log2-trasformed antibody levels (µg/mL), mean values vary across different vaccines. The correlation among antibody levels was stronger between antibodies combined in the same vaccination (Figure S2). No substantial sex differences were observed for most antibody levels, except for measles antibody levels in girls vs. boys (1.06 vs. 0.80; *p* = 0.0008).

**Table 1:** Sociodemographic characteristics of The Programming Research in Obesity, Growth Environment and Social Stress (PROGRESS) study participants included in the analyses. Sociodemographic characteristics were also classified by child sex

Characteristic	Overall population	Girls	Boys	*p*-value^a^
	(*n* = 362)	(*n* = 175, 48%)	(*n* = 187, 52%)	
Child age (years) Mean (SD)	4.75 (0.7)	4.71 (0.72)	4.78 (0.69)	0.3246
Maternal age (years) Mean (SD)	27.78 (5.59)	27.66 (5.78)	27.90 (5.42)	0.5709
Child age (categorical)				
4 years	143 (39%)	75 (43%)	68 (36%)	0.5707
5 years	172 (48%)	77 (44%)	95 (51%)	
6 years	43 (12%)	21 (12%)	22 (12%)	
7 years	4 (1%)	2 (1%)	2 (1%)	
Socioeconomic index (SES)^b^				0.9132
Low	185 (51%)	88 (50%)	97 (52%)	
Medium	140 (39%)	68 (39%)	72 (38%)	
High	37 (10%)	19 (11%)	18 (10%)	
Log2 Antibody levels (µg/mL) Mean (SD)				
Diphtheria	−4.87 (1.09)	−4.83 (1.09)	−4.91 (1.09)	0.3473
Pertussis	−0.75 (1.24)	−0.78 (1.35)	−0.71 (1.13)	0.9836
Tetanus	−2.21 (1.12)	−2.16 (1.13)	−2.25 (1.10)	0.2968
Measles	0.92 (0.82)	1.06 (0.78)	0.80 (0.84)	0.0008
Mumps	1.28 (0.47)	1.29 (0.44)	1.27 (0.50)	0.722
Rubella	2.32 (0.51)	2.39 (0.47)	2.27 (0.53)	0.05452

^a^Sex differences in categorical variables tested using Pearson Chi-Square and Fisher’s exact test, differences in continuous variables tested using Mann Whitney U test

^b^SES is defined using the 13-factor AMAI index

### Individual phthalate metabolite analysis

**Fig. 2:**
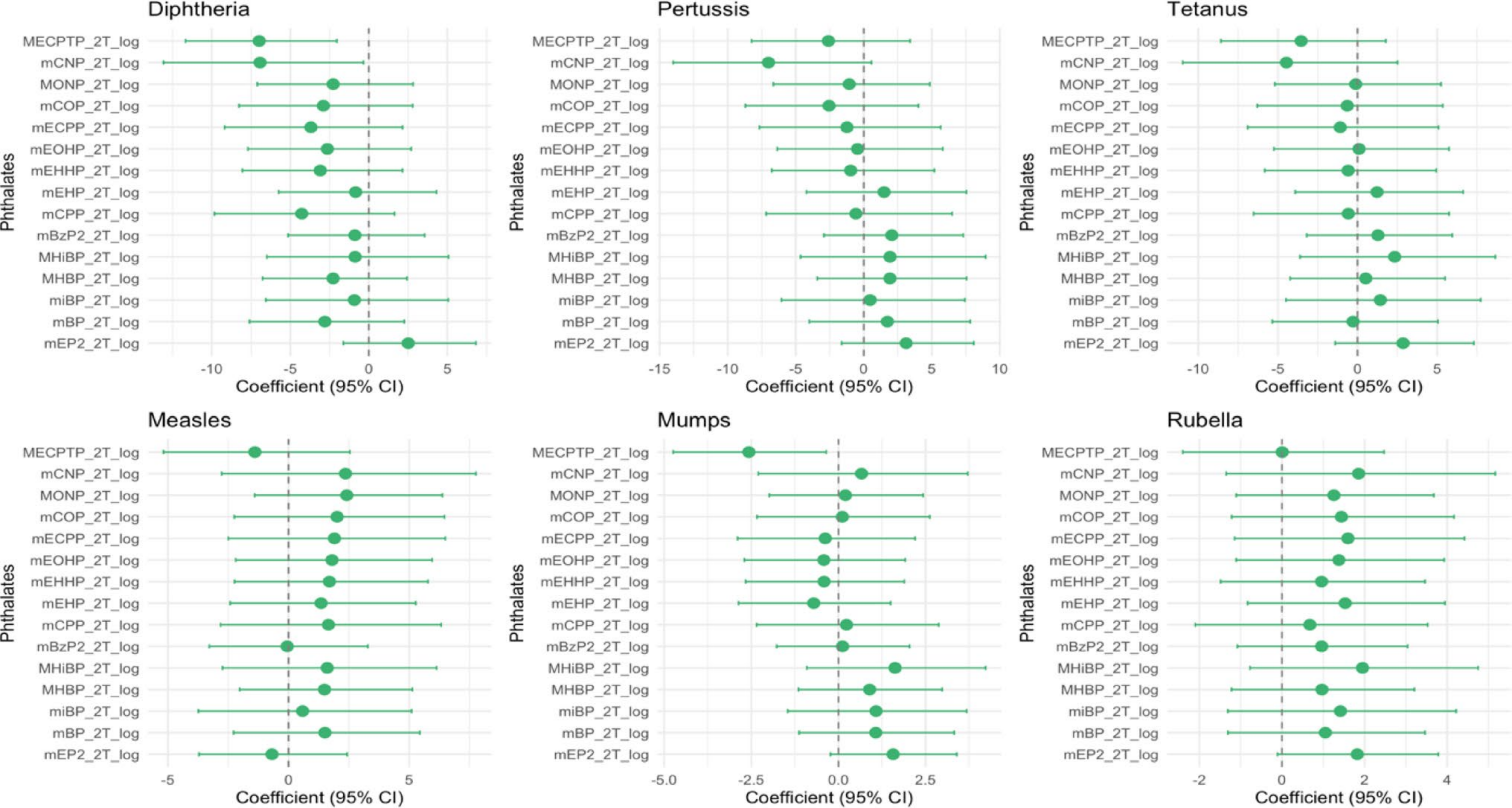
Results from the individual metabolite analyses, assessed in the second trimester of gestation. Forest plot of the associations (coefficient estimates and 95%Confidence Interval (95%CI)) between log-transformed phthalate metabolite concentrations (assessed in the second trimester of gestation) and log2-transformed antibody response to vaccinations in PROGRESS children (at 4-year visits). All results were reported as the percent change (%) in mean antibody levels for a doubling increase of individual phthalate metabolite concentration (i.e. (2^b^ – 1) × 100%, where b represents coefficient of the outcome association with the individual metabolite). All analyses were adjusted for child age, sex, and family socioeconomic status

**Fig. 3:**
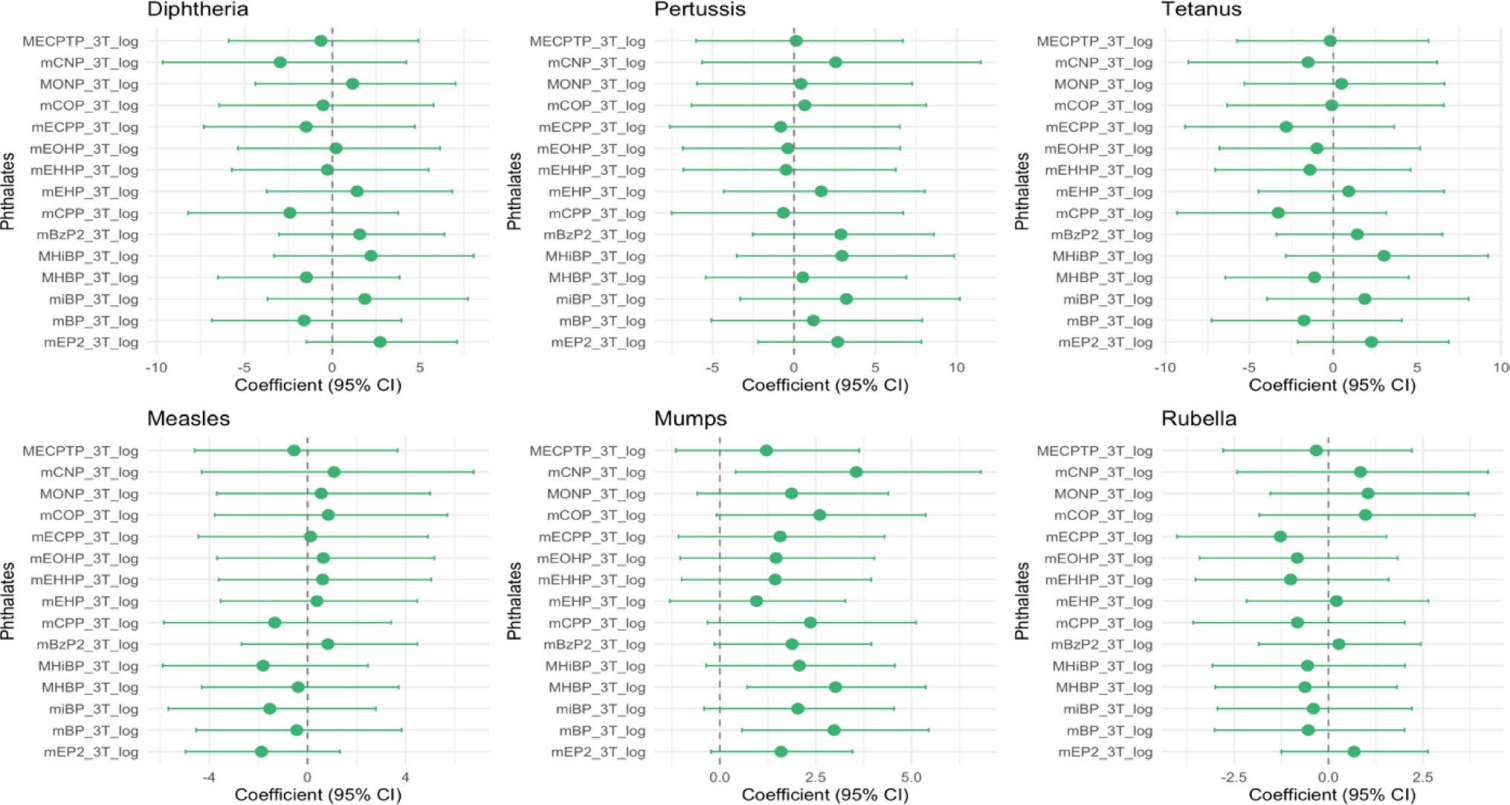
Results from the individual metabolite analyses, assessed in the third trimester of gestation. Forest plot of the associations (coefficient estimates and 95%Confidence Interval (95%CI)) between log-transformed phthalate metabolite concentrations (assessed in the third trimester) and log2-transformed antibody response to vaccinations in PROGRESS children (at 4-year visit). Forest plot of the associations between log-transformed phthalate metabolite concentrations (assessed in the second trimester of gestation) and log2-transformed antibody response to vaccinations in PROGRESS children. All results were reported as the percent change (%) in mean antibody levels for a doubling increase of individual phthalate metabolite concentration (i.e. (2^b^ – 1) × 100%, where b represents coefficient of the outcome association with the individual metabolite). All analyses were adjusted for child age, sex, and family socioeconomic status

In the individual phthalate metabolite regression models, findings varied by metabolite, exposure assessment period, and vaccine-specific antibody (Figs. 2 and 3, Tables S2, S3). Sensitivity analyses examined replacement of concentrations below the LOD with LOD/$\surd\left(2\right)$; no meaningful differences of distributions or associations were observed [18].

*For sample collected during the second trimester*, a doubling increase in MECPTP concentrations was associated with a 7% and 2.5% decrease in diphtheria ($\beta$ = −6.98; 95% CI: −11.68, −2.04) and mumps ($\beta$ = −2.57; 95% CI: −4.74, −0.35) antibody levels, respectively (Fig. 2; Table S2); while a doubling increase in MCNP concentrations was negatively associated with diphtheria antibody concentrations ($\beta$ = −6.93; 95% CI: −13.08, −0.34) (Fig. 2). After multiple testing correction, MECPTP remained statistically significant for diphtheria at 10% FDR (Figs. 2 and S3).

*For sample collected during the third trimester*, a doubling increase in concentrations of MHBP ($\beta$ = 3.01; 95% CI: 0.71, 5.37), mBP ($\beta$ = 2.98; 95% CI: 0.57, 5.45), and mCNP ($\beta$ = 3.56; 95% CI: 0.41, 6.81) showed nominally significant positive associations with mumps antibody concentrations (Figs. 2 and S1; Table S3). None of those associations were significant after correcting for multiple testing (Table S3).

### Quantile G-computational approach results

**Fig. 4:**
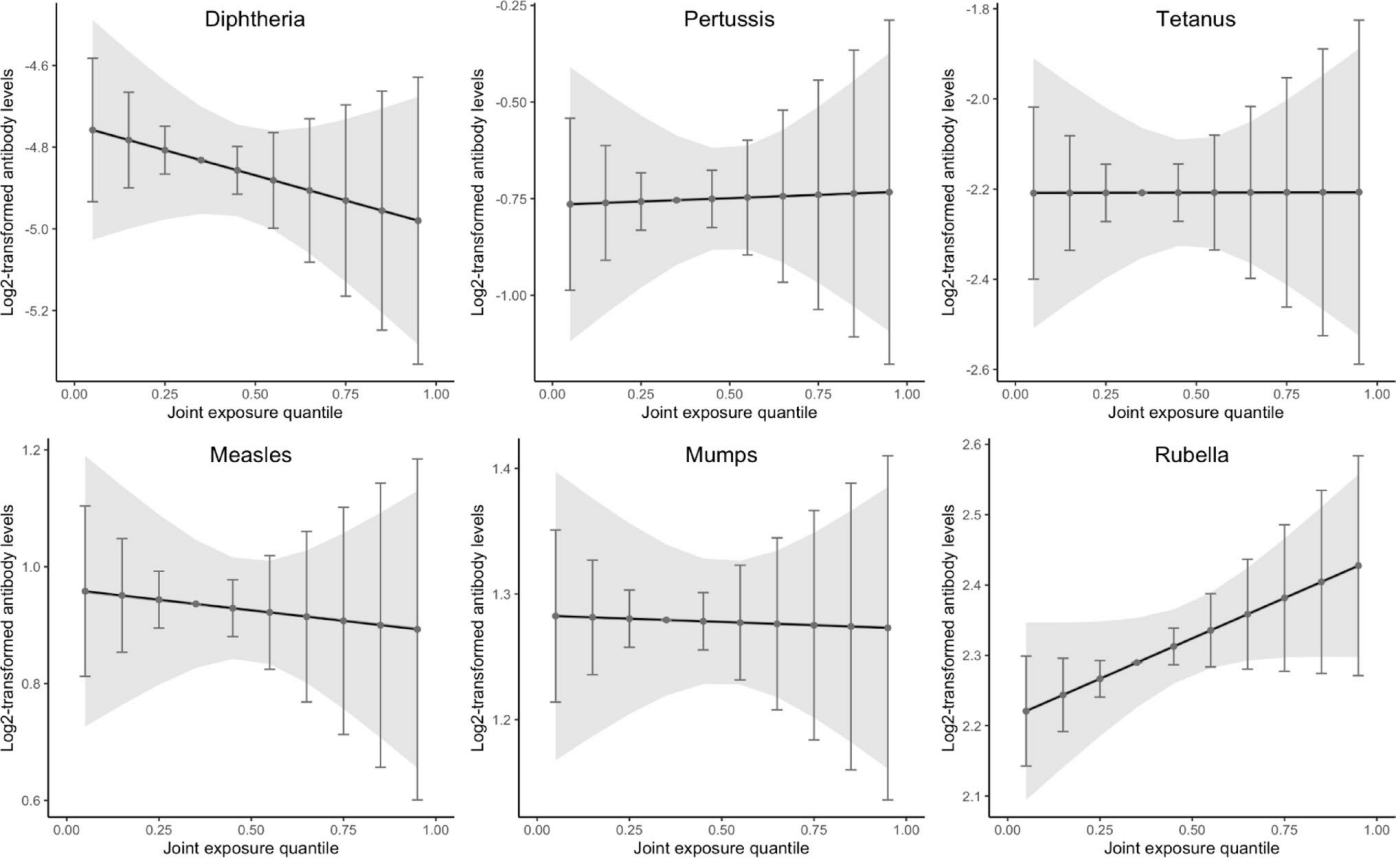
Results from the Quantile G-Computation regressions with metabolites assessed in the second trimester of gestation. Associations between the phthalate metabolite mixture (ranked in deciles and assessed in the 2nd trimester) and log2-transformed antibody response to vaccinations in PROGRESS children (at 4-year visit). All results were reported as change in log2-trasformed antibody levels for a decile increase in the phthalate metabolite mixture. All analyses were adjusted for child age, sex, and family socioeconomic status. Grey ribbons indicate 95%confidence band, while bars indicate pointwise 95% confidence intervals

**Fig. 5:**
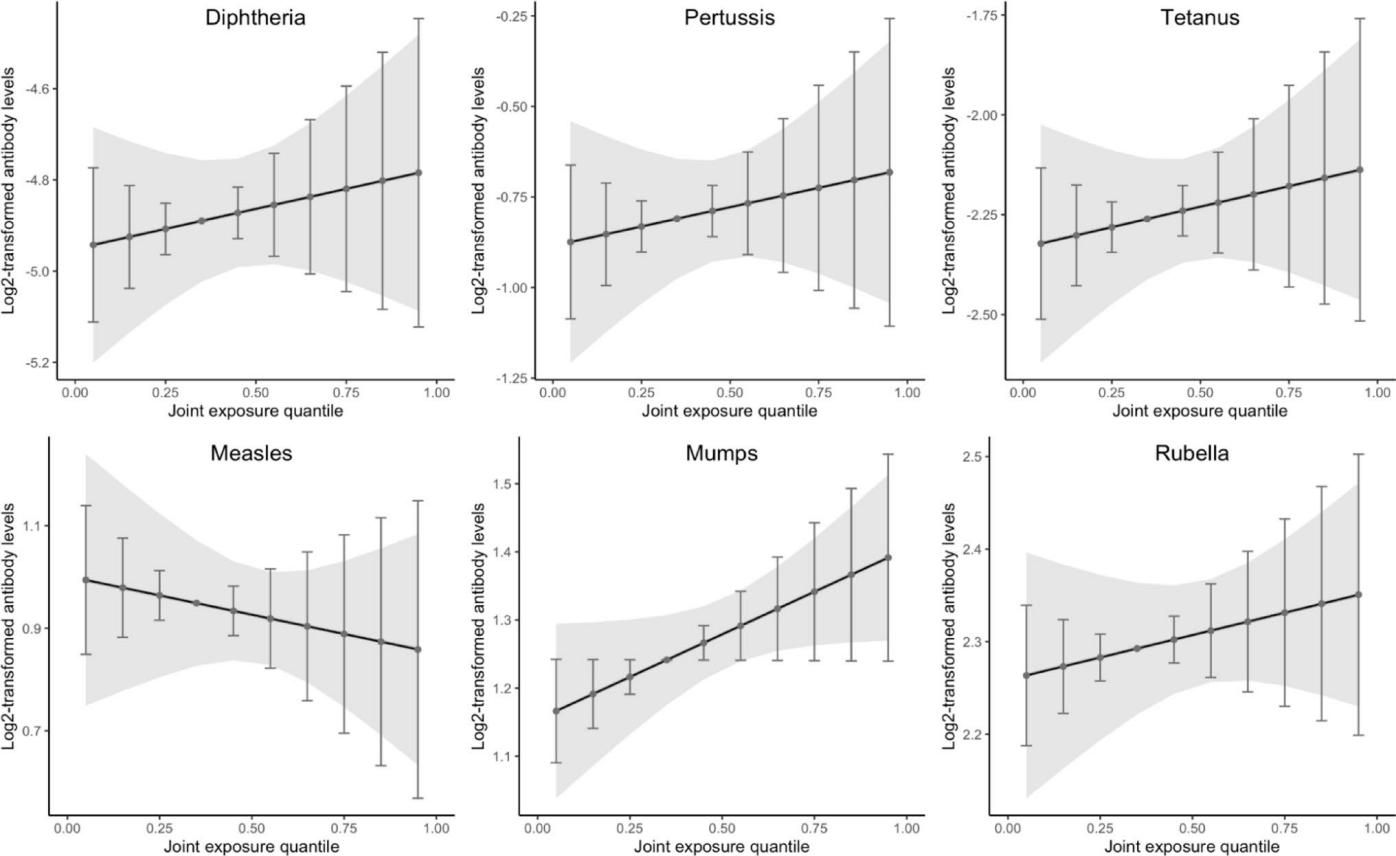
Results from the Quantile G-Computation regressions with metabolites assessed in the third trimester of gestation. Associations between the phthalate metabolite mixture (ranked in deciles and assessed in the 3rd trimester) and log2-transformed antibody response to vaccinations in PROGRESS children (at 4-year visit). All results were reported as change in log2-trasformed antibody levels for a decile increase in the phthalate metabolite mixture. All analyses were adjusted for child age, sex, and family socioeconomic status. Grey ribbons indicate 95%confidence band, while bars indicate pointwise 95% confidence intervals

The mixture analyses assessed in the second trimester showed no statistically significant associations between the overall mixture and antibody concentrations, although some of antibody levels showed a negative direction, such as diphtheria ($\psi$ = −1.70; 95% CI: −5.49, 2.24; *p* = 0.41), measles ($\psi$ = −0.50; 95% CI: −3.42, 2.50; *p* = 0.77), and positive associations with rubella ($\psi$ = 1.61; 95% CI: −0.25, 3.50; *p* = 0.77) (Fig. 4; Table S2). When comparing the third trimester results, only measles retains a negative association with the overall phthalate mixture ($\psi$ = −1.04; 95% CI: −4.08, 2.10; *p* = 0.54), with mumps, diphtheria, pertussis and tetanus indicating a positive direction of association (Fig. 5; Table S2). However, all estimates were very imprecise with wide confidence intervals and none achieving statistical significance.

### Bayesian Kernel machine regression results

**Fig. 6:**
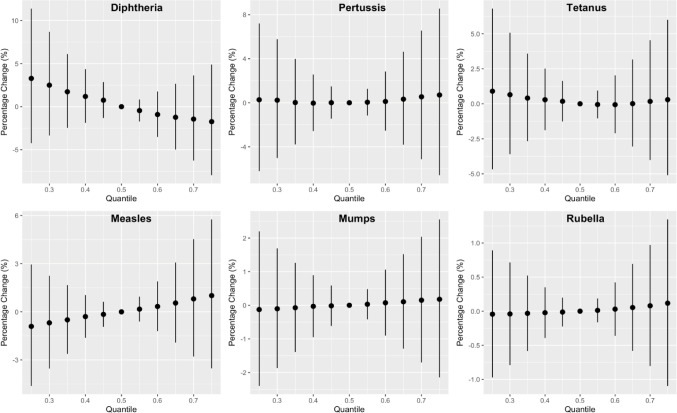
Results from the Bayesian Kernel Machine regressions with phthalate metabolites concentrations measured in the second trimester of gestation. Associations between the non-linear and non-additive phthalate metabolite mixture (assessed in the 2nd trimester) and log2-transformed antibody response to vaccinations (at 4-year visit) in PROGRESS children (i.e., overall effect of the predictors that compares the value of the kernel function when all of predictors are at a particular percentile as compared to when all of them are at their 50th percentile). All results were reported as the percent change (%) in mean antibody levels for a decile increase in the phthalate metabolite mixture (i.e. (2^b^ – 1) × 100%, where b represents coefficient of the outcome association with the phthalate metabolite mixture, respectively). All analyses were adjusted for child age, sex, and family socioeconomic status

**Fig. 7:**
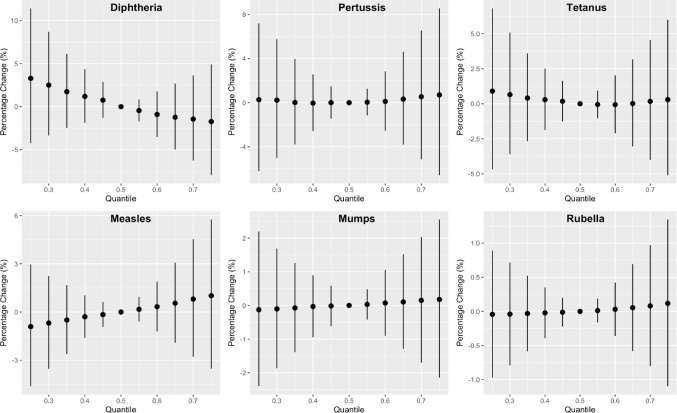
Results from the Bayesian Kernel Machine regressions with phthalate metabolites concentrations measured in the third trimester of gestation. Associations between the non-linear and non-additive phthalate metabolite mixture (assessed in the 3rd trimester) and log2-transformed antibody response to vaccinations (at 4-year visit) in PROGRESS children (i.e., overall effect of the predictors that compares the value of the kernel function when all of predictors are at a particular percentile as compared to when all of them are at their 50th percentile). All results were reported as the percent change (%) in mean antibody levels for a decile increase in the phthalate metabolite mixture (i.e. (2^b^ – 1) × 100%, where b represents coefficient of the outcome association with the phthalate metabolite mixture, respectively). All analyses were adjusted for child age, sex, and family socioeconomic status

The Bayesian Kernel Machine Regression (BKMR) model showed consistency in the directionality of the associations with the quantile g-computation regressions and none of the associations showed a statistically significant associations considering the phthalate metabolite mixtures both at the second and third trimesters (Figs. 6, 7; Table S5). The BKMR regressions suggested that dose-response associations may be present, although the non-linear associations between exposures and the outcomes were minimal (Figure S4); no interactions were identified across exposures (Figures S5-S6).

### Sex-stratified associations

In sex-stratified analysis, MECPTP exposure during the second trimester remained negatively associated, at the nominal significance level, with diphtheria antibody concentrations among boys ($\beta$ = −7.71; 95% CI: −13.91, 1.06, *p* = 0.02) and girls ($\beta$ = −6.56; 95% CI: −4.17, 2.75, *p* = 0.089), although no sex-differences were identified (p-for interaction = 0.728) (Figures S3, Table S2). Most of the associations that were nominally statistically significant in the overall population were more pronounced among boys (Figures S7-S8).

## Discussion

In this study we investigated the associations between gestational phthalate exposures and child serum antibody response to routine vaccinations. Both individual phthalate metabolite associations and mixture associations were evaluated. As phthalates have relatively short biological half-lives, exposures were assessed at two time points during pregnancy to better evaluate the detrimental sensitive window for reduced antibody response. A doubling increase in concentrations of MECPTP during the second trimester of pregnancy was negatively associated with anti-diphtheria antibody levels in PROGRESS children. We did not observe statistically significant associations between phthalate metabolite concentrations during the third trimester and any phthalate metabolite mixtures and child antibody levels.

Previous literature examining this relationship is very limited with inconsistent findings [12, 36]. Only one previous study investigated the role of maternal and pediatric urinary phthalate concentrations and antibody concentrations to DTP and hepatitis B vaccines in a sample of 81 mother-child pairs in Taiwan [12]. Maternal monomethyl phthalate (MMP) and MEHHP concentrations were associated with higher tetanus antibody levels in children at ages 11 and 14 years, respectively [12]. Child MEHHP, MEOHP, and ΣDEHP urinary concentrations at 11 years of age were negatively associated with diphtheria and tetanus antibody levels at 11 and 14 years of age. Additionally, child MnBP, MEHHP, MEOHP, and ΣDEHPm urinary concentrations at 11 years of age were associated with hepatitis B antibody levels at 14 years of age [12]. An additional study investigating the association between concurrent infant DEHP exposure through intravenous infusions (*n* = 15) compared with those not exposed (*n* = 10) and immunoglobulin M (IgM) antibody response to the recombinant hepatitis B vaccine given at birth [36]. In this limited sample, infants exposed to DEHP had enhanced levels of hepatitis B IgM vaccine response [36]. There are clear limitations of these studies including limited control of confounders and small sample sizes. However, there is indication in the prospective epidemiologic study that phthalate exposure assessed at multiple time points may be a better indicator for reduced immune responses given phthalates have a relatively short half-life. The previous studies observed associations with ΣDEHP, MEHHP, MEOHP, MnBP, and MMP. Neither study investigated MECPTP, which suggested potential effects on anti-diphtheria concentrations in the present analysis. Despite initial reports that MECPTP/DEHTP may be less toxic to reproductive tissue, kidneys, and liver [37], new evidence suggests potential associations between MECPTP and behavioral problems in childhood from the PROGRESS cohort [38], and pediatric asthma in the US [39].

Among results of phthalate exposure in the second trimester, boys tended to have associations with antibody levels that were further away from the null, both in positive and negative directions although results were mostly imprecise. Inconsistent differences were observed with phthalate metabolite concentrations in the third trimester and antibody response by sex. Given phthalates have short half-lives in urine and serum of 6 to 64 h [40], investigating gestational phthalate metabolite concentrations with later antibody response in childhood is likely a weak indicator for immune response. Future studies investigating this association should include concurrent phthalate metabolite measures to vaccination and measurement of vaccine response.

In the present analysis, examination of sex-specific effects was exploratory due to limited sample size, marginal differences were observed between boys and girls. The limited previous studies did not investigate differences in the association between phthalate metabolite concentrations and antibody response by sex. However, significant literature has observed sex-differences between phthalate metabolite concentrations and other health outcomes including, but not limited to cognitive function [41], pediatric obesity and cardiometabolic traits [42, 43], play behaviors [44, 45], thyroid function [46], and sexual maturation.[47] These relationships require further investigation.

In mixture analyses imprecise and inconsistent associations were observed between phthalates mixtures in the second and third trimesters with negative and positive associations with antibody response to these routine vaccinations. Although some overall mixtures showed decreasing associations with diphtheria and measles, others showed positive associations with mumps, pertussis, tetanus, and rubella, and others showed a null effect. These relationships varied by trimester of mixture and methods of mixture analysis (Quantile G Computation vs. BKMR). Given these inconsistencies it is unclear how to interpret the relationships between phthalate mixtures and antibody response. Inconsistencies are likely a result of a small sample size leading to significant imprecision. Additionally, urinary phthalate exposures are not indicative of long-term phthalate levels in mothers or children and may not be capturing an appropriate measure or timing of phthalate levels and their potential impact on immune dysfunction measured through antibody response in childhood. Future studies need additional repeated phthalate measures during childhood and near the timing of vaccination to better understand the relationship between these phthalates and antibody response.

Several potential biologic mechanisms could explain associations between phthalates and antibody response to vaccinations in childhood; however these are hypothesized mechanisms based on previous literature which could not be investigated in the present analysis. Phthalates are endocrine disrupting chemicals that can interfere with hormone function and have been associated with immune dysregulation [48–51]. Early-life phthalate exposure has been linked epidemiologically to altered levels of sex and thyroid hormones in children and adolescents, which in turn influence immune function. Gonadal steroid hormone in children modulate B cell and T cell activity and are critical for humoral immunity, including vaccine-induced antibody responses [52–55]. Epidemiologic evidence has observed phthalate exposure in early-life to have inverse associations with testosterone levels in childhood [56–58], and to alter estradiol, luteinizing hormone, thyroid stimulating hormone and other hormone levels in children and adolescents [59]. Additionally, there has been evidence of sexually dimorphic changes in vaccine response. An epidemiologic study of 1,324 school-aged Spanish children reported 2% greater MMR vaccine antibody levels in girls (*p* < 0.001), compared with boys [60]. An additional study of young adults observed 50% higher post-vaccination antibody titers to measles in girls, compared with boys [61]. In addition to hormonal pathways, gestational phthalate exposure may influence offspring vaccine-induced antibody responses through interconnected maternal–fetal mechanisms. During pregnancy, phthalates have been associated with increased maternal oxidative stress and altered inflammatory and endocrine signaling, which can affect placental function and fetal immune development [62–64]. Consequently, prenatal exposure could have long-term effects on immune function including reduced antibody production or altered responsiveness following routine childhood vaccinations [12].

The primary strength of our study lies in the application of several statistical approaches, including both individual chemical analysis and mixture regressions, to pinpoint the gestational phthalate metabolites most strongly associated with antibody response. Understanding the most impactful phthalates can provide valuable insights for guiding intervention efforts aimed at reducing these exposures. An additional strength of the present study is its longitudinal design allowing for multiple exposure measurements, multiple covariate assessments, and assessment of a long-term outcome of antibody response. Two urine samples were collected and analyzed (one in the 2nd and one in the 3rd trimester of pregnancy) to better assess exposure within trimesters of pregnancy and to characterize a sensitive window of exposure. The present study can adequately assess temporality aiding with causal inference, however, given the relatively short biological half-life of phthalates, concurrent assessments of exposures and outcomes may be more appropriate to elucidate this relationship. Compared with the very limited existing literature, the present study included a much larger sample. Lastly, given the extensive data collection as part of the PROGRESS study, multiple potential confounders and risk factors for antibody response were considered, however, none appeared to be related to phthalate metabolites and antibody response in the study sample. Given strong a priori evidence that child age, sex, and socio-economic status are potential confounders, these were adjusted for in the present analyses.

Several limitations must be noted as well. Although the sample size in the present analysis was larger than those used in previous literature, observed effect sizes were very small, the present analysis is likely under-powered leading to non-significant associations and imprecise confidence limits between mixtures and antibody response and synergistic relationships among metabolites may have been missed. The present study did not include postnatal exposures, which are likely more indicative of concurrent immune response to vaccinations. Additionally, only two prenatal urinary phthalate concentrations are examined during the second and third trimester of pregnancy. Phthalate concentrations in urine are indicative of short-term phthalate exposure in the previous 12–48 h [15]. For this reason, these single exposure measures may not be appropriate measures of phthalate exposure to the fetus during gestation. However, women who have chronic and consistent exposure to phthalates through daily use of personal care products, living environments, regular dietary sources and others may have relatively stable phthalate measures overtime. Within-person variability in phthalate concentrations has been shown to be high with repeated spot urine samples generally showing moderate reproducibility for some phthalates [15, 16]. Ultimately, single urine sample may over or underestimate exposure levels and multiple samples over days or weeks are a better indicator of average exposure. Future studies should consider more frequent repeated measures while also considering phthalate exposure at time of vaccination and at time of antibody response measurement and the critical period of exposure to phthalate and the potential effect on vaccines may be during the immune response itself and not during the time of immune development during gestation. The acute phthalate measurement will likely lead to an exposure misclassification of maternal phthalate exposure; however, the misclassification should be non-differential by antibody response endpoints and will likely attenuate our results towards the null. Additionally, only a single assessment of childhood antibody response levels was measured, continuing work is measuring antibody response at additional time points. However, in this population most participants had serum antibody levels at or above the clinical correlation of protection leading to reduced variability of the outcome. Future research directions should consider the inclusion of interaction and quadratic terms in the analysis and the evaluation of alternative algorithms. This work would require a larger sample size than that in the present analysis. Future studies should extend our findings by evaluating longitudinal assessments of immune-related outcomes and clinical outcomes. A recent systematic review examining the impact of toxic metals of immune dysfunction observed limited evidence of a link between reduced immune biomarkers (i.e., antibody response, T cell counts, B cell counts, other immunoglobulins, etc.), making it difficult to understand how large of a reduction in these biologic endpoints is clinically meaningful [65].

In the present analysis, we did not have information on vaccination adherence. The MMR and DTP vaccines are required vaccines for children to attend school and are routinely performed at 4 years of age. Given our population had a mean age of 4.7 years for antibody measurement we concluded the assumption that most of these children had already received MMR and DTP vaccines. Additionally, these vaccine-preventable diseases are not common in Mexico, indicating any antibody levels observed are due to vaccination and not community exposure. Additionally, time since vaccination is an important indicator of antibody response, although we do not believe it will be associated with phthalate concentrations during pregnancy and would not be a confounder in the present analysis. Lack of information on vaccine adherence and timing might impact our precision and appropriate interpretation for public health implications but should not impact our effect estimates observed. The present study aimed to assess the impact on vaccine-specific IgG antibody response in early childhood related to gestational phthalate concentrations, future work should consider vaccination and potential vaccine effectiveness in a larger cohort for clearer public health implications of these relationships.

There may be residual confounding by maternal nutritional status as certain diets may increase risk of phthalate exposure and may impact fetal and infant immune development. In addition, other factors including vaccine adherence/timing, breastfeeding status, overall maternal health, pregnancy complications, and pediatric infection history may be potential confounders for the associations as they can influence childhood immune system responsiveness and they can reflect chronic childhood phthalate exposures. Unfortunately, lack of data on these potential confounders limited our ability to identify the entire causal structure between phthalate exposures and childhood antibody response to routine vaccinations and future studies should focus on causal approaches including these potential confounders. Lastly, this work was done in a population of Hispanic children residing in Mexico City. Although Mexico follows a very similar vaccination schedule to that of the United States—making the populations living in the U.S. and in Mexico comparable in terms of immunization outcomes—their phthalate metabolite distributions and lifestyle characteristics may differ and may not be comparable and not even generalizable to other populations. Phthalate exposures have been reported to be higher among individuals living in Mexico or among Mexican immigrants living in the USA compared to the general U.S. population. However, these higher exposure levels may enhance the ability to increase the statistical power to detect associations even with a smaller sample size, compared to populations with lower exposure levels. Given these limitations, this work, although novel, is exploratory and requires significant additional research with longitudinal assessment of the outcomes to better elucidate the associations between phthalate exposure and antibody response to vaccinations and their trajectories.

Despite these limitations, this study adds to the limited body of literature investigating the potential associations between phthalate exposures in early life and childhood vaccine response. The limitations and findings of the present study can help inform future epidemiologic studies in their development and determination of methods and data collection.

## Conclusions

In summary, a doubling increase in concentrations of a single phthalate metabolite (MECPTP) during the second trimester of gestation were negatively associated with anti-diphtheria antibody levels in Hispanic children. We observed inconsistent and non-statistically significant associations between phthalate metabolite mixtures and child antibody levels including negative associations with measles and diphtheria, and positive associations with mumps, tetanus, and rubella. The association between prenatal phthalate levels and antibody response remains unclear. However, there is some suggestion that MECPTP, a replacement phthalate, may be associated with lower diphtheria antibody response. These potential associations and mechanisms need to be more thoroughly investigated in additional longitudinal studies among diverse populations.

## Supplementary Information

Below is the link to the electronic supplementary material.

Supplementary Material 1

Supplementary Material
